# Supplementary material for: Integrated Transcriptome and Metabolome Analysis Identifies Key Genes Regulating Maize Tolerance to Alkaline Stress
Source: Int J Mol Sci. 2025 Oct 31;26(21):10632. doi: 10.3390/ijms262110632 (PMC12607767; doi:10.3390/ijms262110632)
Supplement: Supplementary file 1 [file ijms-26-10632-s001.zip › ijms-3886547-supplementary.pdf]

Supplementary Material (Figure S1-S14)

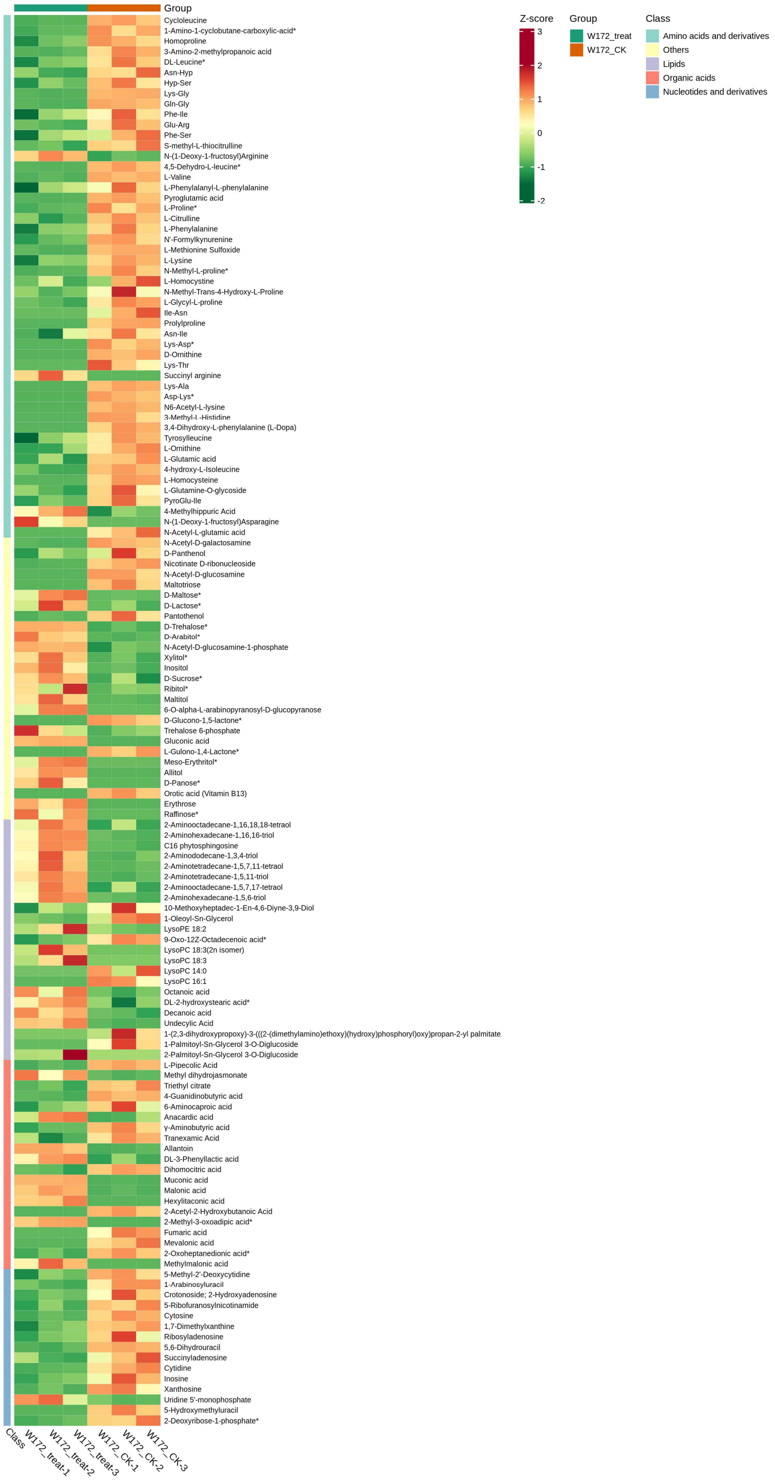

Figure S1 Differential accumulation of metabolites clustering heatmap of W172_treat vs W172_CK.

The normalized signal intensities of the metabolites are visualized as a color spectrum, where different colors represent different relative levels (red indicates high levels, green indicates low levels).

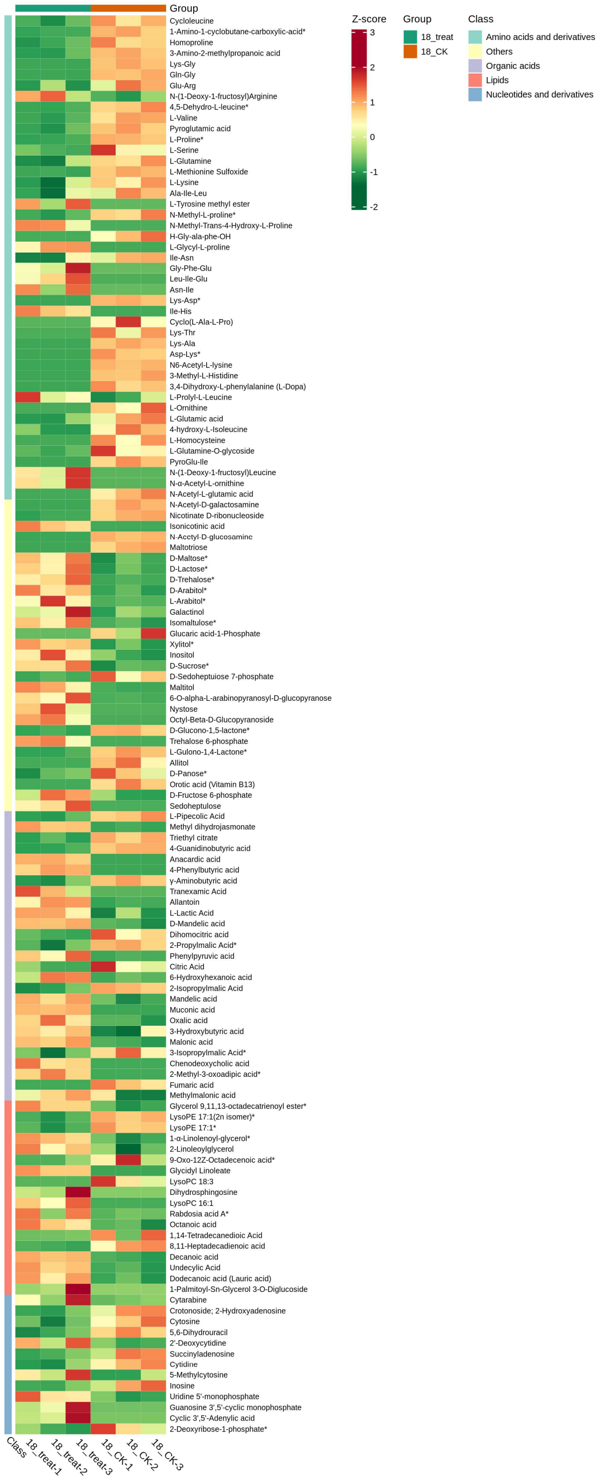

Figure S2 Differential accumulation of metabolites clustering heatmap of 18_treat vs 18_CK. The normalized signal intensities of the metabolites are visualized as a color spectrum, where different colors represent different relative levels (red indicates high levels, green indicates low levels).

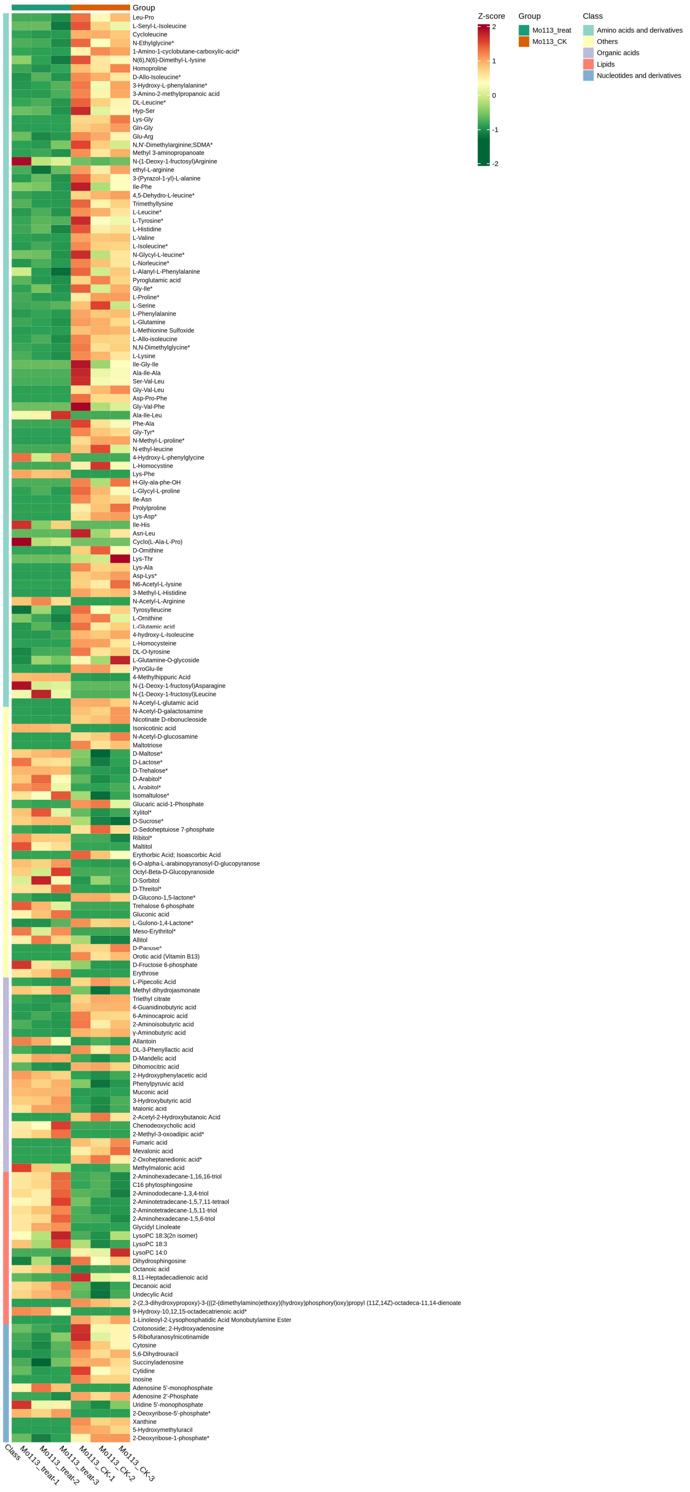

Figure S3 Differential accumulation of metabolites clustering heatmap of Mo113_treat vs Mo113_CK.

The normalized signal intensities of the metabolites are visualized as a color spectrum, where different colors represent different relative levels (red indicates high levels, green indicates low levels).

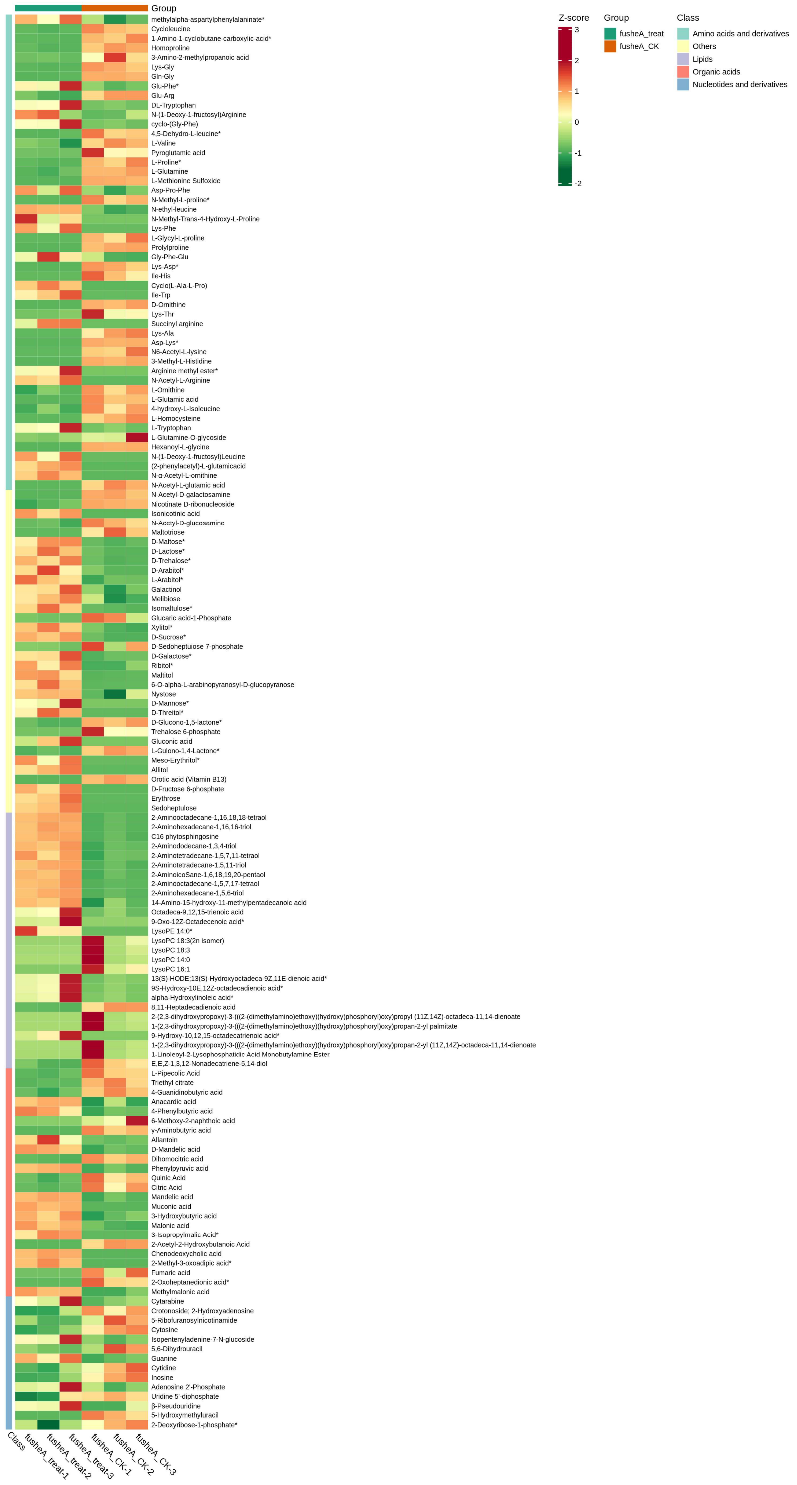

Figure S4 Differential accumulation of metabolites clustering heatmap of fusheA_treat vs fusheA_CK.

The normalized signal intensities of the metabolites are visualized as a color spectrum, where different colors represent different relative levels (red indicates high levels, green indicates low levels).

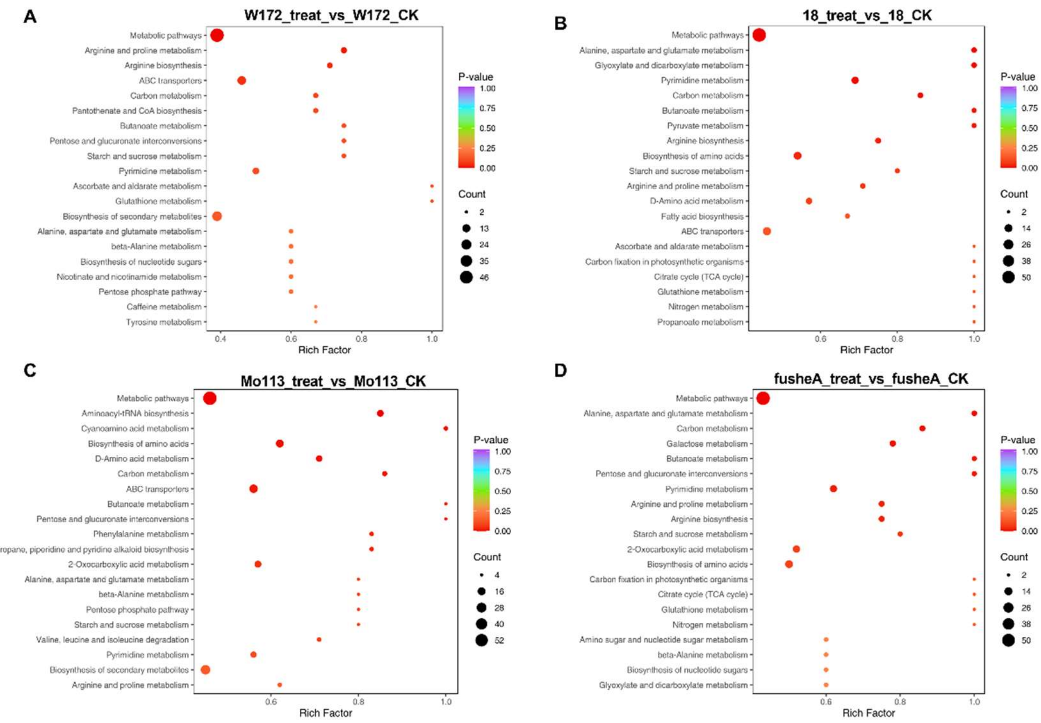

Figure S5 KEGG pathway enrichment analysis of the metabolome in four maize inbred lines after alkali treatment. (**A**) W172_treat_vs_W172_CK. **(B)** 18_treat_vs_18_CK. (**C**) Mo113_treat_vs_Mo113_CK. (**D**) fusheA_treat_vs_fusheA_CK.

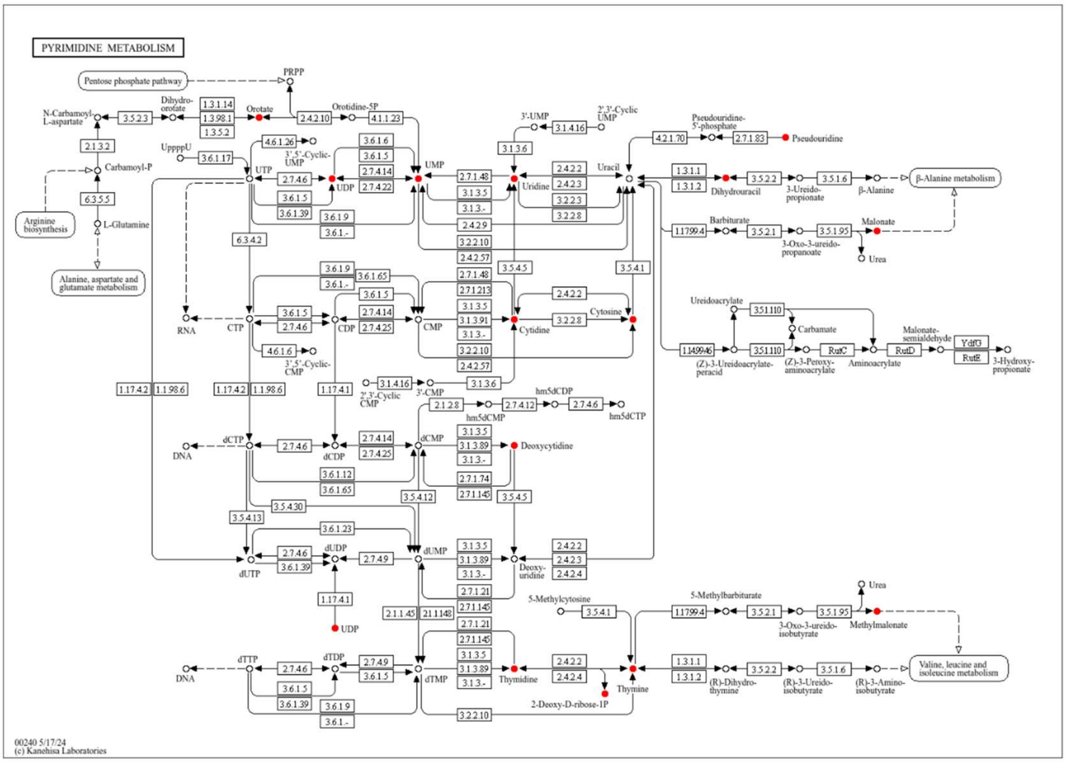

Figure S6 Schematic diagram of pyrimidine metabolic pathways. The metabolites highlighted in red were detected in this study.

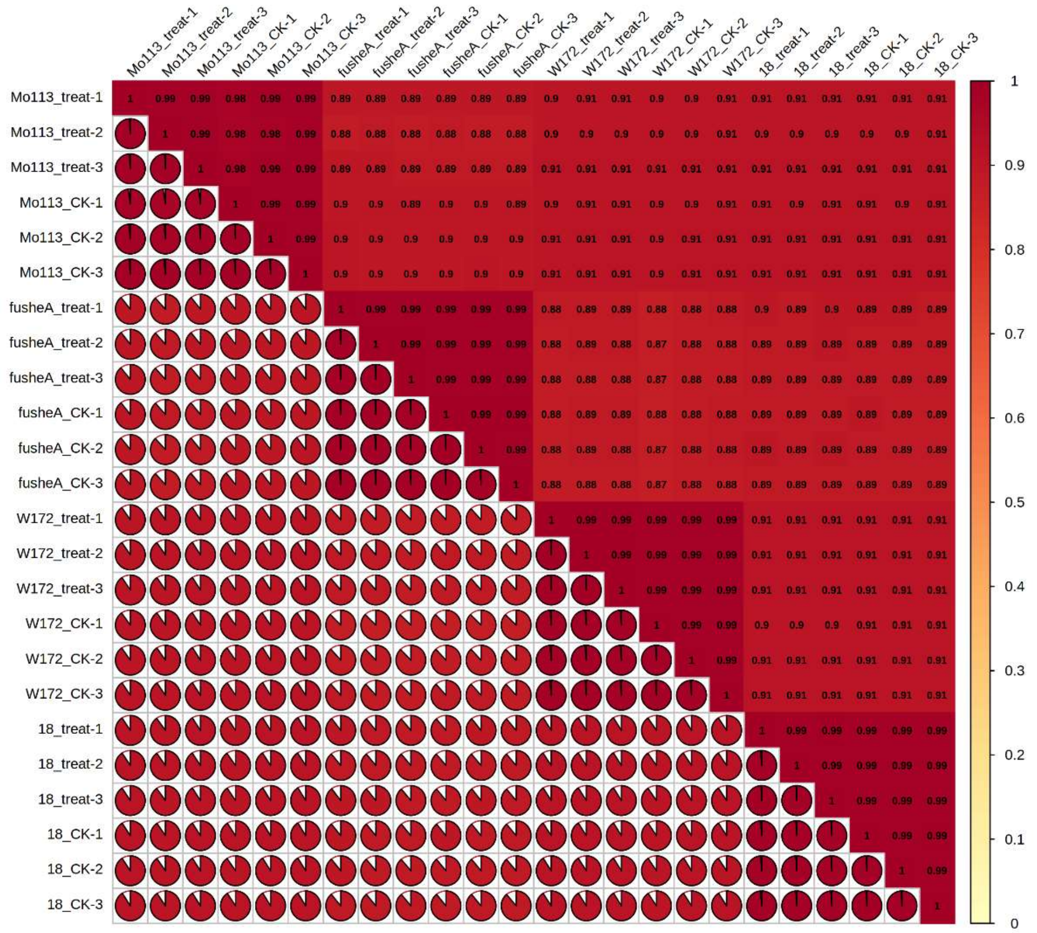

Figure S7 Pearson correlation between transcriptome samples.

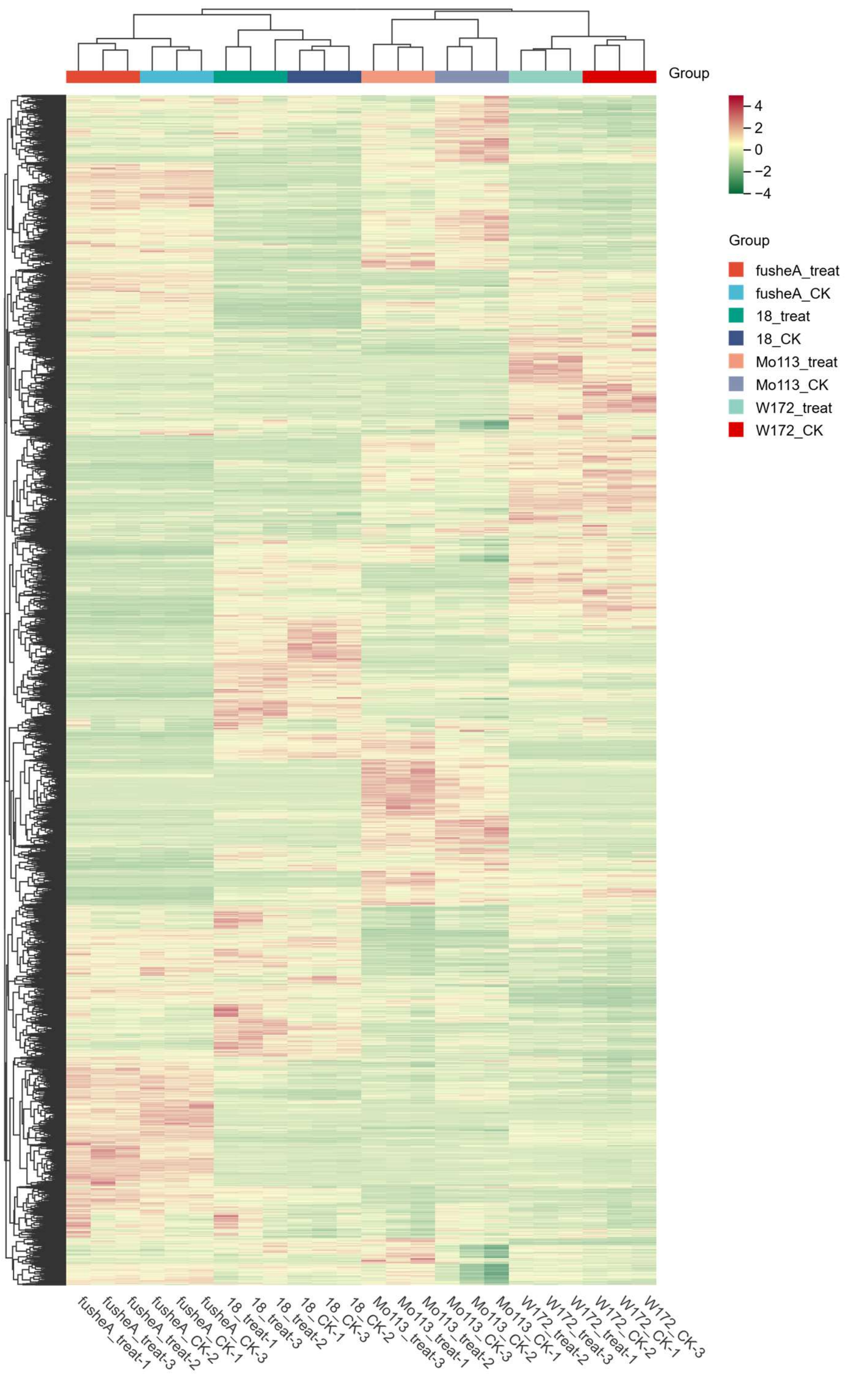

Figure S8 Hierarchical clustering heatmap of differentially expressed genes

The normalized signal intensities of the metabolites are visualized as a color spectrum, where different colors represent different relative levels (red indicates high levels, green indicates low levels).

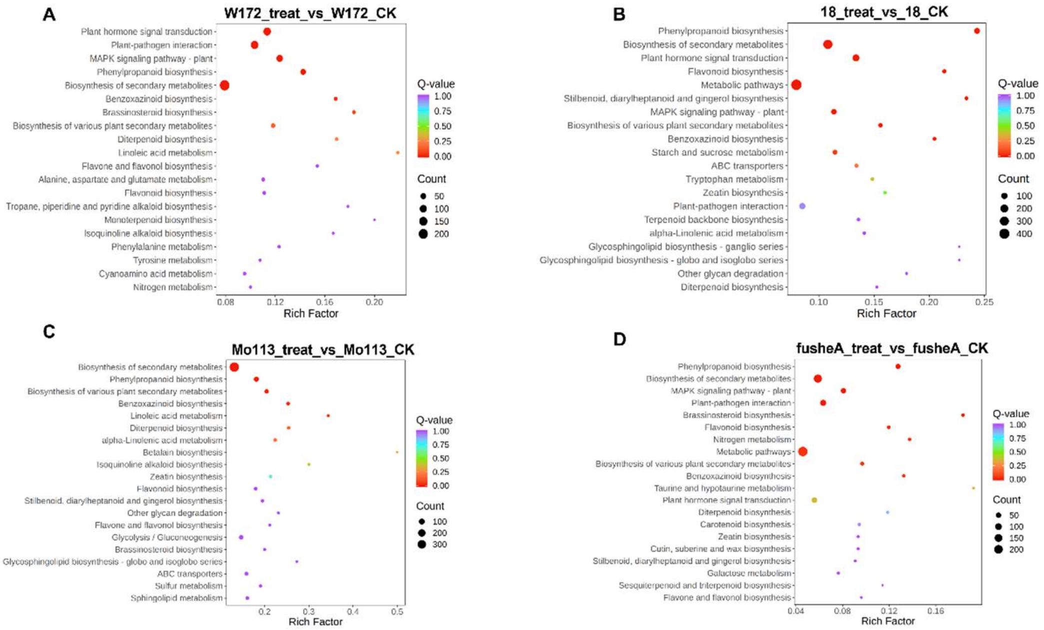

Figure S9 KEGG pathway enrichment analysis of the transcriptome in four maize inbred lines after alkali treatment. (**A**) W172_treat_vs_W172_CK. **(B)** 18_treat_vs_18_CK. (**C**) Mo113_treat_vs_Mo113_CK. (**D**) fusheA_treat_vs_fusheA_CK.

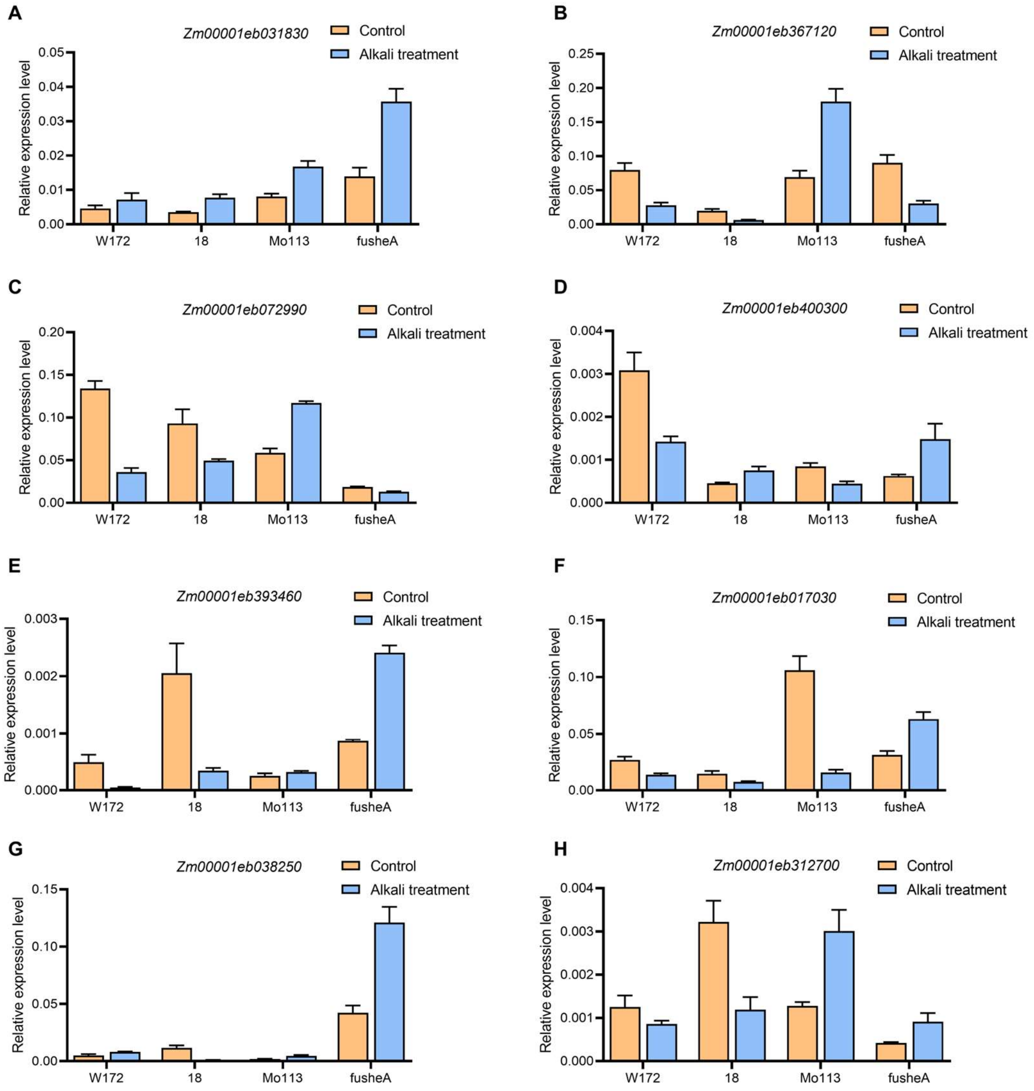

Figure S10 Verification of the reliability of transcriptome data by RT-qPCR. During the verification process, each inbred line material contains three replicates.

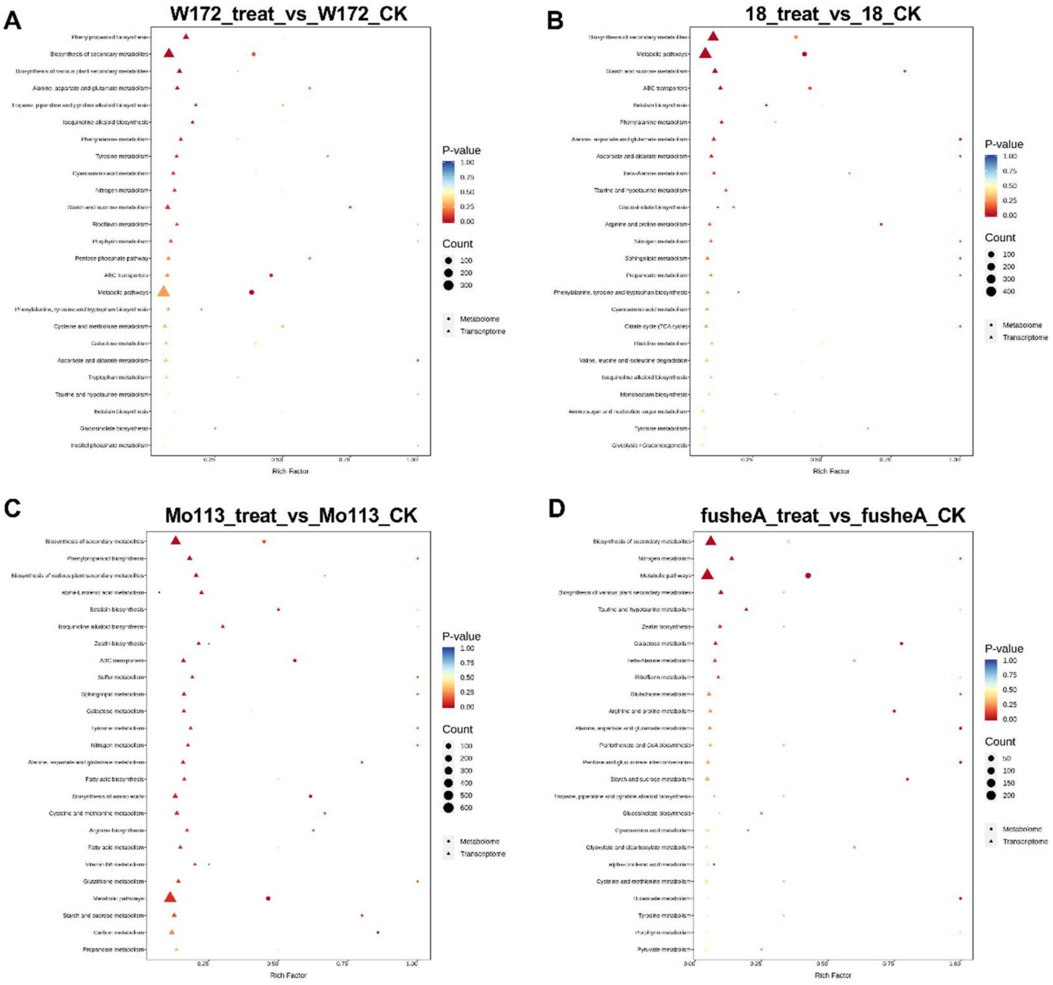

Figure S11 Transcriptome & metabolome KEGG pathways Co-enrichment(A) W172_treat vs W172_CK common KEGG enrichment bubble. **(B)** 18_treat vs 18_CK common KEGG enrichment bubble. **(C)** Mo113_treat vs Mo113_CK common KEGG enrichment bubble. **(D)** fusheA_treat vs fusheA_CK common KEGG enrichment bubble. (The horizontal axis denotes the enrichment factors (Diff/Background) of this pathway across different omics, while the vertical axis indicates the names of the KEGG pathways. The gradient from red to yellow to blue reflects the variation in the significance level of enrichment, ranging from high to medium to low, as represented by the p-value. The shape of the bubbles corresponds to distinct omics types, and the size of the bubbles signifies the number of differential metabolites or genes, with larger sizes indicating greater numbers.)

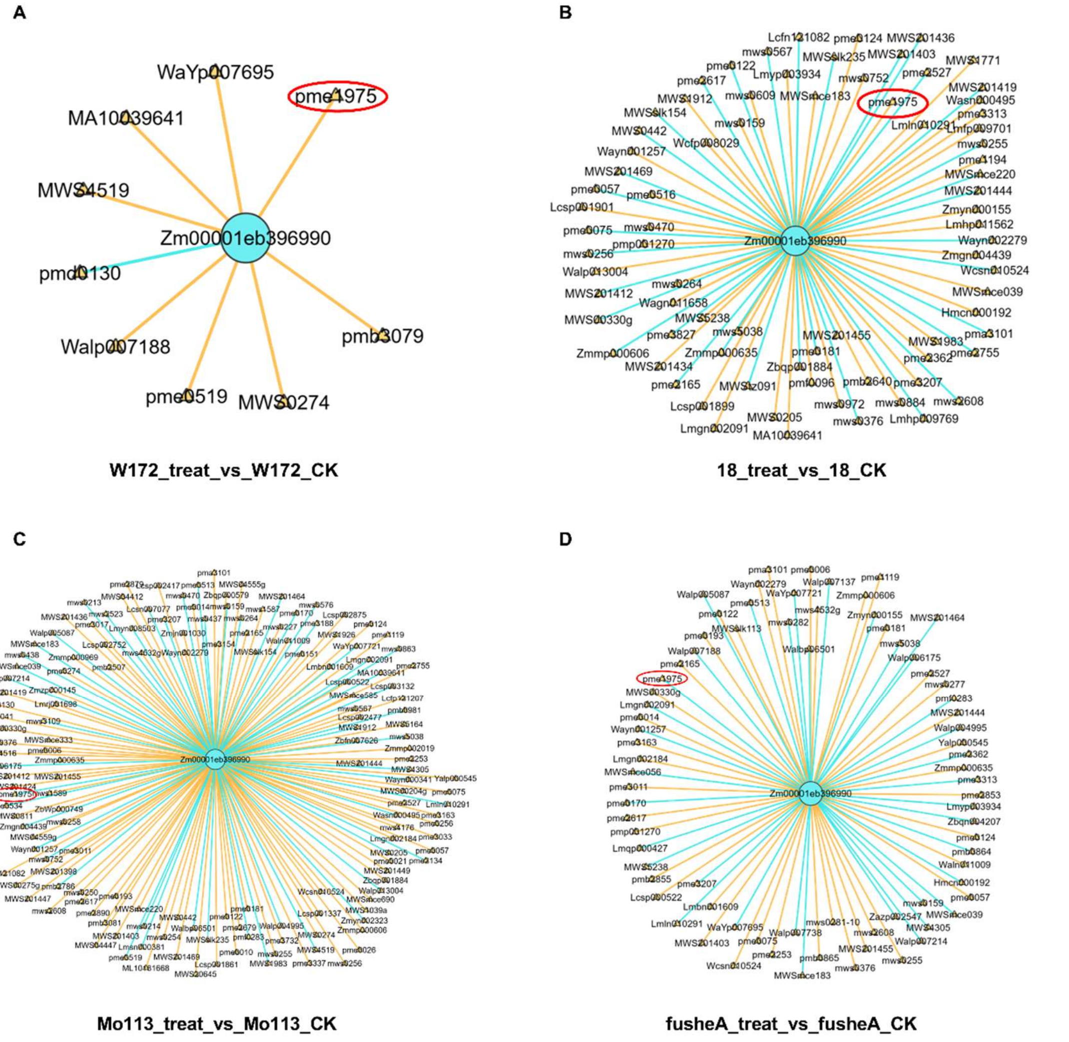

Figure S12 Correlation analysis of differentially expressed metabolites and candidate genes in four maize inbred lines after alkali treatment. **(A)** W172_treat vs W172_CK. **(B)** 18_treat vs 18_CK. **(C)** Mo113_treat vs Mo113_CK. **(D)** fusheA_treat vs fusheA_CK. The blue line represents the negative correlation and the yellow line represents the positive correlation. The yellow triangles represent differentially expressed metabolites, and the blue circles represent candidate genes.

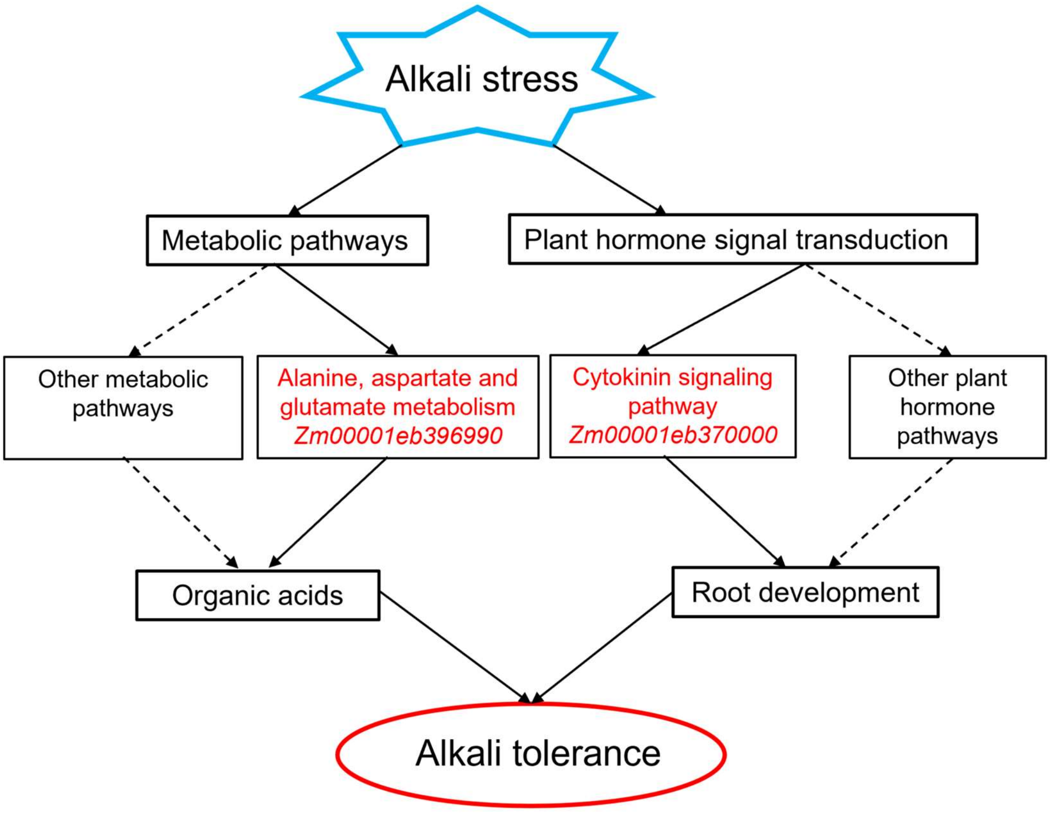

Figure S13 Schematic diagram of maize transcriptional regulation and metabolic pathways in response to alkaline stress."

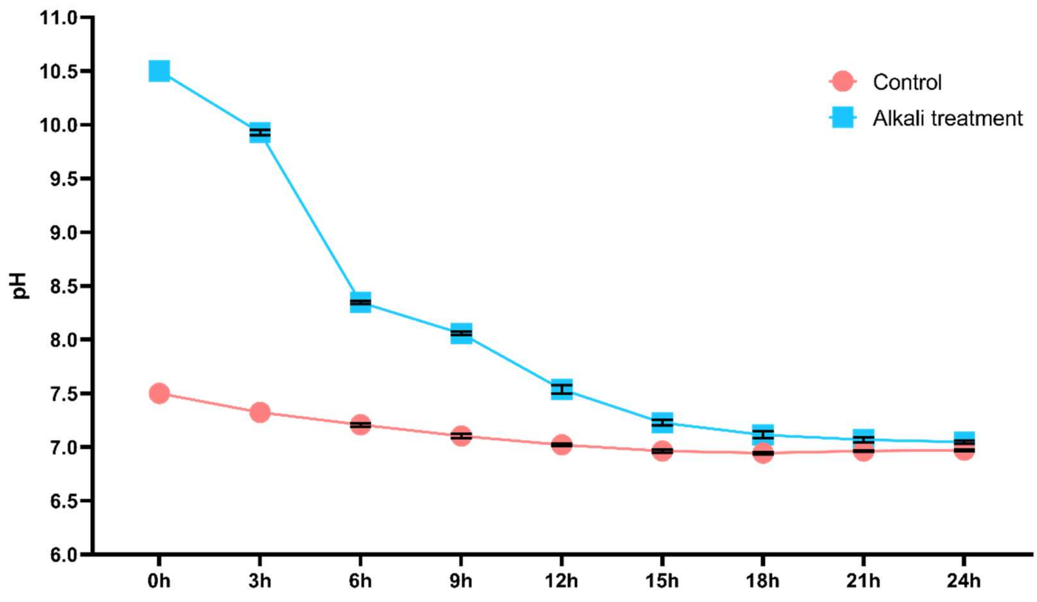

Figure S14 pH curve of B73 inbred line treated with deionized water and alkali for 24 h. The control group was treated with deionized water. Three repeated measurements were conducted at each time point.
